# PCR Detection of HHV8 DNA in the Saliva of Removable Denture Wearers Compared to Dentate Cases in Shiraz, South of Iran

**DOI:** 10.1155/2020/9358947

**Published:** 2020-04-15

**Authors:** Reza Derafshi, Jannan Ghapanchi, Fahimeh Rezazadeh, Mohammad Hasan Kalantari, Amir Mosahebi Naeeni, Mitra Farzin, Afagh Moattari

**Affiliations:** ^1^Department of Prosthodontics, School of Dentistry, Shiraz University of Medical Sciences, Shiraz, Iran; ^2^Department of Oral and Maxillofacial Medicine, School of Dentistry, Shiraz University of Medical Sciences, Shiraz, Iran; ^3^Oral and Dental Disease Research Center, Department of Oral and Maxillofacial Medicine, School of Dentistry, Shiraz University of Medical Sciences, Shiraz, Iran; ^4^Student Research Committee, School of Dentistry, Shiraz University of Medical Sciences, Shiraz, Iran; ^5^Department of Microbiology, Shiraz University of Medical Sciences, Shiraz, Iran

## Abstract

**Result:**

In the denture wearers, HHV8 DNA was detected in 11 cases. Two of the controls amplified HHV8 DNA. Fisher's exact test demonstrates a significant difference between virus infection and using removable dentures (*p* = 0.015).

**Conclusion:**

Our findings suggested that HHV8 detection could be associated with use of denture.

## 1. Introduction

The oral microbial flora is highly diverse; it mainly consists of bacteria and fungi. It is clear that changing of normal flora can promote oral disease process because these microorganisms play important roles in preventing colonization of pathogenic agents [1, 2].

Special anatomical and physiological properties of the oral cavity make it a desirable location for pathogen growth. The saliva droplet oropharyngeal secretion spread the microorganisms through sneezing, coughing, speaking, or breathing [3, 4]. Due to significant relationship between oral microbiota and numerous systemic diseases, in addition to role of general and oral hygiene maintenance in colonization of microorganisms associated with these infections, the impact of evaluating these pathogens is more clear [5]. Oral health is a reflection of one's general health [3, 6], and its status declines with age [7]. It has been recognized that the oral status of older people can impact on their general health, quality of life, and well-being [8].

Adults used complete or partial dentures in order to replace missing teeth. Use of removable dentures may cause some variations in the oral microbial flora. In many cases, this condition tends to cause denture-related stomatitis [9].

Human herpes virus 8 (HHV8) is an oncogenic virus that can cause Kaposi's sarcoma (KS) and lymphoproliferative diseases such as primary effusion lymphoma and multicentric Castleman disease [10].

In a study of Kaposi's sarcoma case body fluids, HHV8 was found in the saliva more than genital secretions and the titers in saliva were higher than semen [11]. A research showed that HHV8-associated lymphoma was detected in two old HIV negative cases [12]. As many studies showed, removable dentures may act as reservoir for bacterial pathogens that can cause serious infections [13, 14]. Up to now, none of the studies evaluate the accumulation of the viruses in the saliva of Iranian population, especially old cases that may be hazardous for them. Thus, this study was designed to evaluate HHV8 in the saliva of patients with removable dentures.

## 2. Materials and Methods

### 2.1. Ethical Statement

This cross-sectional study was carried out in accordance with the guidelines of the Declaration of Helsinki as revised in Edinburgh (1975). The study protocol was approved by the Ethics Committee of Shiraz University of Medical Sciences, Shiraz, Iran. The written informed consents were obtained from participants for sample collection, and in unable cases, verbal consent was obtained. Patients were informed about the nature of the study.

### 2.2. Participants

In a cross-sectional study from March-July 2019, saliva samples were collected from 50 denture wearers as a case group and 50 ages and gender matched dentate subjects as a control. Whole-mouth saliva, parotid saliva, buccal, and palatal exfoliates were collected and processed for HHV8-DNA amplification as described by Al-Otaibi et al. [15]. Saliva samples were collected at the Shiraz dentistry school on the day of the clinical examination; subjects were asked not to consume food or liquids for 1 hour prior to sample collection. The collected samples, which were accumulated for five minutes, hold in sterilized plastic tubes that were subsequently stored at −20°C. The saliva samples were provided from 58 males and 42 females. The case groups were edentulous and wearing their present full dentures for at least 7 years and all were admitted to the Department of Prosthodontics, Dental School, Shiraz University of Medical Sciences, Shiraz, Iran. The sampling method was convenience sampling. The exclusion criteria were systemic conditions that could affect the oral flora and any use of antivirals and mouth washes before the study. Eighteen subjects were suffering from hypertension or cardiovascular disease, and the rest were healthy. Their systemic diseases were under control, and they used related drugs routinely. All controls were admitted to other departments of dental school for routine dental care. Dentate subjects had at least 8 teeth or more. The participants in both groups were married and nearby all of them brushed their teeth or washed their dentures at least once a day.

### 2.3. DNA Extraction

The detection of HHV8 DNA by the polymerase chain reaction (PCR) assay has been the primary technique used to detect the virus in different locations [16].

DNA was extracted from saliva by Geneall (Germany) kit. HHV8-DNA amplification was performed by applying a nested PCR protocol [17]. The initial amplification was conducted using the primers showed in Table 1.

The PCR was performed in a 30 *μ*l volume reaction mix and produced an amplicon of approximately 220 base pair (bp). The constituents of the mix were 3 *μ*l of Taq polymerase buffer (1%) (Ampliqon, Austria ®), 1.5 *μ*l of MgCl2 (3 mM) (Taq-Gen MgCl2 50 nM, Ampliqon, Austria), 2.4 *μ*l of dNTPs (dNTP mixture®, 100 nM solution, Amresco®), 3 *μ*l of Taq polymerase, 3 *μ*l of each primer, and 13.9 *μ*l of nuclease-free water (Nuclease-Free Water, Promega®). The PCR program was conducted in the thermocycler by the following steps: denaturing 94°C for 5 min, 34 cycles (94°C for 1 min + 60°C for 1 min + 72°C for 1 min), and final extension at 72°C for 1 min.

Nested PCR was used to find HHV8 DNA in saliva [18]. For nested PCR, 0.5 *μ*l of first-round product was transferred to 29.5 *μ*l of an identical PCR mix, but containing second-round primers with the same concentration as the first round. PCR conditions were the same as for first-round PCR. Positive and negative controls were included in each run. PCR amplicons were then electrophoresed through a 2% agarose gel and visualized through a transilluminator (Vilber Lourmat®, France). For a more accurate result, each test repeated 3 times.

A confirmatory real-time PCR assay was done to amplify highly conserved ORF73 genes of HHV8. The quality real-time PCR primers and probe sequences were 5′-CCGAGGACGAAATGGAAGTG-3′, 5′-GGTGATGTTCTGAGTACATAGCGG-3′, and [5′-(6FAM) ACAAATTGCCAGTAGCCCACCAGGAGA (TAMRA)-3, respectively. The amplification was performed in a 20 *μ*l reaction mixture with a quantitative real-time PCR (BioFact, Korea). The reaction mixture contained 3 *μ*l of DNA solution, 2 *μ*l of 10x TaqMan buffer, 0.4 *μ*l of each primer (20 *μ*M), 0.4 *μ*l of probe (10 *μ*M), and 0.4 *μ*l of *Taq* DNA polymerase enzyme.

Following, the program of qRT-PCR at 95°C, 45 cycles (15 s at 95°C and 1 min at 65°C) were performed with an rotor-gene 6000 (*Corbett* Life Science). Briefly, fluorescence measurements were taken every 7 s, and a threshold cycle (CT) value for each sample was calculated by determining the points at which the fluorescence exceeded a threshold limit (10 times the standard deviation of the baseline). Standard curves were established with the PCR product of HHV8, which included both the target sequence of HHV8 primer. PCR products were analyzed by 1.5% neutral agarose gels with SYBR safe stain (YTA, Iran). The gels were visualized under UV light with a transilluminator (UV-GENTM; Bio-Rad Laboratories) and cut out the band with a razor blade. After cutting out the band, follow the procedure for DNA fragment purification using DNA purification kit (Favorgen Biotech, Taiwan), following the manufacturer's procedure. Nucleic acid concentration was measured by the NanoDrop Instrument (Thermo Scientific™ 2000). Then, copy number of HHV8 template DNA was calculated based on the following formula:

(1) $$\begin{array}{c} \text{Number of copies}\left(\text{molecules}\right)=\frac{\left[X\left(\text{ng}\right)*6.022*1023\right]}{\left[\text{length}\left(\text{bp}\right)*\text{conversion factor}*660\right]}*42, \end{array}$$

where *X* is nucleic acid concentration, length of DNA template is the base pairs (bp) of PCR product, Avogadro's constant (6.022^∗^1023) represents the number of molecules in 1 mole, conversion factor is (1^∗^109), and the average mass of 1 bp of dsDNA is the average mass of 1 bp of dsDNA is 660 g/mole. For standardization of the real-time PCR detection assay, ten-fold dilution series of the HHV8 genomic DNA yielded nine points on a curve spanning 101 to109 parasites. Standard curve was calculated automatically by plotting the Ct values against each known standard and then calculated the linear regression line of this curve. The control (HHV8 PCR product) was diluted in water from 109 to 1 copy number per sample. Each sample was submitted to the HHV8 real-time PCR, and amplifications were repeated five times for each dilution. A standard curve of the CT values plotted against the logarithm of the copy number was constructed. HHV8 quantification proved to be linear over a wide range (from 10 to 109 copies per well). The detection rate was 100% when the copy number was ≥10 copies per well and was 75% for 1 copy per well, which is an agreement with the values that can be estimated from the Poisson probabilities. The amplification yield and detection rates were comparable when standard dilutions were submitted.

The positive control consisted of a clinical sample and as a control for cross-contamination; a sample consisting of distilled water was also subjected to the DNA extraction procedure, and the resulting extract was amplified in duplicate.

Samples were considered negative if the CT values exceeded 45 cycles. In addition, several conditions had to be fulfilled for experimental validation.

### 2.4. Statistical Analysis

The statistical analysis for case-control groups was carried out by using SPSS software version 15. The chi-square test was used to compare the positive and negative case regarding age, gender, marital status, educational level, and labour status. Fisher's exact test also used to correlate the presence of HHV8 in both groups. Statistically, a significant difference was considered when *p* < 0.05.

## 3. Results

All demographic findings of participants were recorded. The frequency of HHV8 in relation to gender in different groups is presented in Table 2.

**Figure 1:**
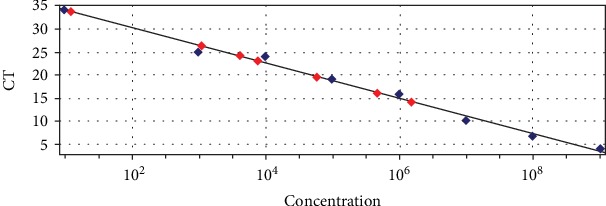
Standard curve of HHV8 RT-qPCR. The standard curve was generated by platting the mean CT value versus the eight-fold serial dilution of DNA standard over a range of concentration from 10^8^ copies per/*μ*l 10^1^ copies/*μ*l.

**Figure 2:**
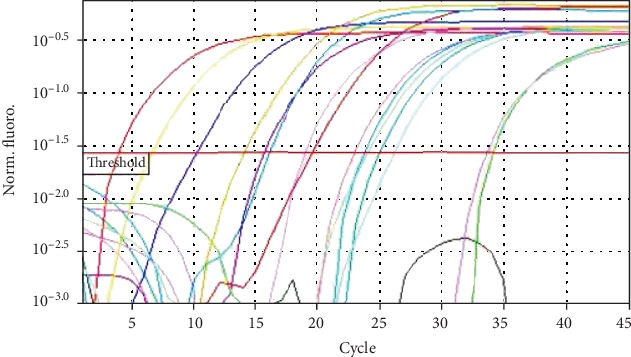
The amplification curve forHHV8 RT- qPCR.

The mean age of the patients was 70.6 ± 10.2. The age range was 60-99 years. The mean age of females and males was 64.14 ± 5.42 and 65.47 ± 8.35, respectively. There was no statistically significant association between the underlying diseases in the control and the case group (hypertension, cardiovascular disease), (*p* > 0.05). In the denture wearers, HHV8 DNA was detected in 8 cases by nested PCR and 9 by real-time PCR. None of the controls amplified HHV8 DNA in the nested method, but using real-time showed 1 positive case. Results show that quantification was linear over serial dilutions. The obtained standard curve had a slope of -3.46; R2 and efficiency percent were 0.997 and 98.31, respectively, which showed a suitable standard curve for quantifying Figure 1. The melt curve of SYBR green qPCR products was shown in Figure 2. The result of real-time PCR showed that HHV8 was detected in 9 cases and 1 control sample. The mean ± SD of HHV8-positive sample copy numbers (9 sample case and 1 sample control) were showed in Table 3.

**Figure 3:**
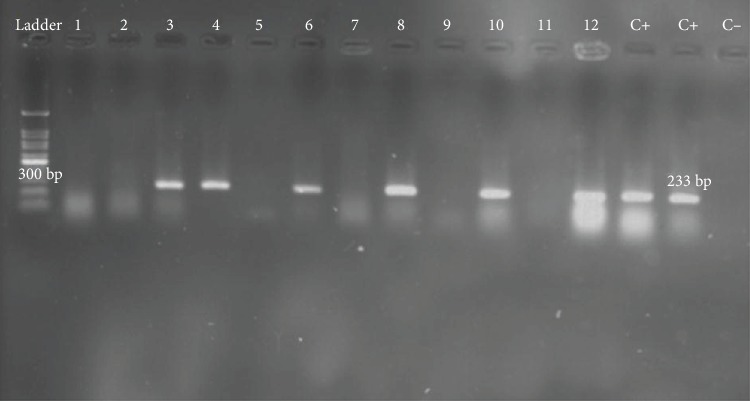
Electrophoresis band.

Chi-square test demonstrates a significant difference between virus infection and using removable dentures (*p* = 0.003). The gel electrophoresis band of nested PCR is shown on Figure 3.

## 4. Discussion

The present study assessed the HHV8 of old age participants wearing dentures without oral lesions and compared them to dentate cases. To the best of our knowledge, this is the first study to evaluate HHV8 load in the saliva of elderlies. We found this hazardous virus in saliva of 9 denture wearers, and that is a dangerous alert for them. The present study does not take into account the bacterial detection in dentures. In a case report, two French old patients infected with HHV8 suffered from serious AIDS-unrelated primary effusion lymphoma [12]. Fortunately, all participants in this study were healthy and without clinical oral lesions.

The oral flora may be changed in debilitating disease, aging, and also by using denture [19–21]. The existing teeth have an important effect on the overall oral bacteria composition in comparison to the total edentulous subjects [22]. Noncommensal bacteria were detected in the saliva of denture wearers [23]. Alterations of the oral cavity condition in reaction to an intraoral prosthesis may cause lack of saliva accessibility and tongue-associated mechanical cleansing [24]. Furthermore, dentures can harbor mixed kinds of bacterial biofilm [25, 26]. 18 kinds of bacteria were isolated from denture plaque that can induce respiratory infection in elderly [19, 27]. In the present study presence of HHV8 in denture user also confirm this important issue.

HHV8 can transmit via nonsexual route. In Uganda, HHV8 seroprevalence increased up to 49% among cases aged ≥50 years [28]. This finding increased the concern of susceptibility of medical problems in the denture user's relatives.

A higher percentage of human herpes viruses were detected in saliva of subjects with Peri-implantitis [29]. HHV8 is detected in the saliva and the clinically healthy oral mucosa of HIV negative immune compromised cases. The oral epithelium looks to reserve the virus and simplify its replication [30]. Current research did not demonstrate a significant difference between both groups regarding gender, marital status, educational level, and labour status (*p* > 0.05), but there was a significant relation between age and amplification of HHV8. Same result was reported by another study which found the elevated antibody titer in older subjects [31].

This finding may be related to increased chance of sexual, nonsexual route of transmission. Serological and molecular researches showed that HHV8 can be widely scattered in the human being and, similar to other herpes viruses, it has the ability of latent infectivity in tissues and body fluids, until recurrence [32, 33].

According to the results of this study, we found significant differences in the amplifying of HHV8 in saliva of denture users than nonusers (*p* = 0.015). The current research also showed that denture should be considered as a favorite reservoir of colonization and maybe subsequent infection in elderly. It seems that proper denture hygiene, use of natural antiviral [34], and local antibacterial agents can reduce numerous microorganisms' accumulation in the mouth.

All participants washed their teeth or dentures at least once a day, so oral hygiene maintenance did not differ between both groups. Further studies focused on oral hygiene (frequency, route) on subjects with similar dentition are suggested in order to evaluate the relation between oral hygiene and salivary HHV8 load. Furthermore, regarding our limitation in sampling (convenience), randomized study will be suggested for future researches.

## 5. Conclusion

In conclusion, a significant difference between presence of HHV8 and using removable denture was shown, and our findings suggested that HHV8 detection could be associated with use of denture. Further studies using a large sample population should investigate the presence of different herpesviridae in saliva samples of denture wearers.

## Figures and Tables

**Table 1:** The primer of HHV8.

Primer	Sequence 5′➔3′
HHV8	*AGC CGA AAG GAT TCC ACC AT*
	*TCC GTG TTG TCT ACG TCC AG*

**Table 2:** Demographic data of patients and controls.

Groups	Patients (denture wearer)	Control (dentate)
Demographic data		
Age	70.6 ± 10.2	65.8 ± 8.6
Gender (female/male)	21/29	22/28
Educational level		
Primary school	13	11
High school	37	39
Marital status		
Single	0	0
Married	50	50
Labour status		
Retired	28	36
Self-employment	11	6
Housewife	11	8
Presence of HHV8	8	0

**Table 3:** The copy number of HHV8 in case and control.

Sample	Copy number of HHV8
Case	
P1	4.2 × 10^5^
P2	2.4 × 10^4^
P3	2.0 × 10^4^
P4	1.2 × 10^4^
P5	1.7 × 10^2^
P6	6.1 × 10^1^
P7	8.2 × 10^3^
P8	1.8 × 10^4^
P9	3.0 × 10^5^
Control	
C1	1.5 × 10^1^

## Data Availability

The data used to support the findings of this study are available from the corresponding author upon request.

## References

[1] Aas J. A., Paster B. J., Stokes L. N., Olsen I., Dewhirst F. E. (2005). Defining the normal bacterial flora of the oral cavity. *Journal of Clinical Microbiology*.

[2] Ghabanchi J., Zibaei M., Afkar M. D., Sarbazie A. (2010). Prevalence of oral Entamoeba gingivalis and Trichomonas tenax in patients with periodontal disease and healthy population in Shiraz, southern Iran. *Indian Journal of Dental Research*.

[3] Li X., Kolltveit K. M., Tronstad L., Olsen I. (2000). Systemic diseases caused by oral infection. *Clinical Microbiology Reviews*.

[4] Rezazadeh F., Shahbazi F., Andisheh-Tadbir A. (2017). Evaluation of salivary level of IL-10 in patients with oral lichen planus, a preliminary investigation. *Comparative Clinical Pathology*.

[5] Dahlén G., Blomquist S., Carlen A. (2009). A retrospective study on the microbiology in patients with oral complaints and oral mucosal lesions. *Oral Diseases*.

[6] Rezazadeh F., Moshaverinia M., Motamedifar M., Alyaseri M. (2016). Assessment of anti HSV-1 activity of aloe vera gel extract: an *in vitro* study. *Journal of Dentistry*.

[7] Castrejón-Pérez R. C., Borges-Yáñez S. A., Gutiérrez-Robledo L. M., Ávila-Funes J. A. (2012). Oral health conditions and frailty in Mexican community-dwelling elderly: a cross sectional analysis. *BMC Public Health*.

[8] Pinke K. H., Freitas P., Viera N. A., Honório H. M., Porto V. C., Lara V. S. (2016). Decreased production of proinflammatory cytokines by monocytes from individuals presenting Candida-associated denture stomatitis. *Cytokine*.

[9] Sunil M., Reid E., Lechowicz M. J. (2010). Update on HHV-8-associated malignancies. *Current Infectious Disease Reports*.

[10] Koelle D. M., Huang M.-L., Chandran B., Vieira J., Piepkorn M., Corey L. (1997). Frequent detection of Kaposi's sarcoma-associated herpesvirus (human herpesvirus 8) DNA in saliva of human immunodeficiency virus-infected men: clinical and immunologic correlates. *Journal of Infectious Diseases*.

[11] Pellett P. E., Spira T. J., Bagasra O. (1999). Multicenter comparison of PCR assays for detection of human herpesvirus 8 DNA in semen. *Journal of Clinical Microbiology*.

[12] Boulanger E., Hermine O., Fermand J. P. (2004). Human herpesvirus 8 (HHV-8)-associated peritoneal primary effusion lymphoma (PEL) in two HIV-negative elderly patients. *American Journal of Hematology*.

[13] Le Bars P., Kouadio A. A., N'goran J. K., Badran Z., Soueidan A. (2015). Relationship between removable prosthesis and some systemics disorders. *The Journal of Indian Prosthodontic Society*.

[14] Przybyłowska D., Mierzwińska-Nastalska E., Swoboda-Kopeć E., Rubinsztajn R., Chazan R. (2016). Potential respiratory pathogens colonisation of the denture plaque of patients with chronic obstructive pulmonary disease. *Gerodontology*.

[15] Al-Otaibi L. M., Ngui S. L., Scully C. M., Porter S. R., Teo C. G. (2007). Salivary human herpesvirus 8 shedding in renal allograft recipients with Kaposi's sarcoma. *Journal of Medical Virology*.

[16] Whitby D., Boshoff C., Hatzioannou T. (1995). Detection of Kaposi sarcoma associated herpesvirus in peripheral blood of HIV- infected individuals and progression to Kaposi's sarcoma. *The Lancet*.

[17] Leao J. C. (1999). *Studies into the In Vivo Interactions between Human Immunodeficiency Virus and Human Herpesvirus 8*.

[18] Cardemil C. V., Parashar U. D., Hall A. J. (2017). Norovirus infection in older adults: epidemiology, risk factors, and opportunities for prevention and control. *Infectious Disease Clinics*.

[19] Moshaverinia M., Rezazadeh F., Dalvand F., Moshaverinia S., Samani S. (2014). The relationship between oral lichen planus and blood group antigens. *World Journal of Medical Sciences*.

[20] Campisi G., Chiappelli M., de Martinis M. (2009). Pathophysiology of age-related diseases. *Immunity & Ageing*.

[21] Razak P. A., Richard K. J., Thankachan R. P., Hafiz K. A., Kumar K. N., Sameer K. (2014). Geriatric oral health: a review article. *Journal of International Oral Health: JIOH*.

[22] O’Donnell L. E., Robertson D., Nile C. J. (2015). The oral microbiome of denture wearers is influenced by levels of natural dentition. *PLoS One*.

[23] Derafshi R., Bazargani A., Ghapanchi J., Izadi Y., Khorshidi H. (2017). Isolation and identification of nonoral pathogenic bacteria in the oral cavity of patients with removable dentures. *Journal of International Society of Preventive & Community Dentistry*.

[24] Vahabi S., Salman B. N., Rezazadeh F., Namdari M. (2014). Effects of cyclosporine and phenytoin on biomarker expressions in gingival fibroblasts of children and adults: an in vitro study. *Journal of Basic and Clinical Physiology and Pharmacology*.

[25] Shi B., Wu T., McLean J. (2016). The denture-associated oral microbiome in health and stomatitis. *mSphere*.

[26] Koopmans A. S. F., Kippuw N., de Graaff J. (1988). Bacterial involvement in denture-induced stomatitis. *Journal of Dental Research*.

[27] Sumi Y., Miura H., Michiwaki Y., Nagaosa S., Nagaya M. (2007). Colonization of dental plaque by respiratory pathogens in dependent elderly. *Archives of Gerontology and Geriatrics*.

[28] Mbulaiteye S. M., Goedert J. J. (2011). *Human Herpesvirus 8 Seropositivity in Rural Uganda: Maturation of Sero-Epidemiological Studies*.

[29] Marques Filho J. S., Gobara J., da Silva Salomao G. V. (2018). Cytokine levels and human herpesviruses in saliva from clinical periodontal healthy subjects with Peri-implantitis: a case-control study. *Mediators of Inflammation*.

[30] Triantos D., Horefti E., Paximadi E. (2004). Presence of human herpes virus-8 in saliva and non-lesional oral mucosa in HIV-infected and oncologic immunocompromised patients. *Oral Microbiology and Immunology*.

[31] Freitas R. B., Freitas M. R., Linhares A. C. (2002). Prevalence of human herpesvirus 8 antibodies in the population of Belém, Pará, Brazil. *Revista do Instituto de Medicina Tropical de São Paulo*.

[32] Schulz T. F., Sheldon J., Greensill J. (2002). Kaposi's sarcoma associated herpesvirus (KSHV) or human herpesvirus 8 (HHV8). *Virus Research*.

[33] Martin J. N., Ganem D. E., Osmond D. H., Page-Shafer K. A., Macrae D., Kedes D. H. (1998). Sexual transmission and the natural history of human herpesvirus 8 infection. *New England Journal of Medicine*.

[34] Ghapanchi J., Moattari A., Tadbir A. A., Talatof Z., Shahidi S., Ebrahimi H. (2011). The in vitro anti-viral activity of honey on type 1 herpes simplex virus. *Australian Journal of Basic and Applied Sciences*.

